# Sex differences in total cholesterol of Vietnamese adults

**DOI:** 10.1371/journal.pone.0256589

**Published:** 2021-08-20

**Authors:** Nga Thi Thu Tran, Christopher Leigh Blizzard, Khue Ngoc Luong, Ngoc Le Van Ngoc Truong, Bao Quoc Tran, Petr Otahal, Mark R. Nelson, Costan G. Magnussen, Tan Van Bui, Velandai Srikanth, Thuy Bich Au, Son Thai Ha, Hai Ngoc Phung, Mai Hoang Tran, Michele Callisaya, Seana Gall

**Affiliations:** 1 Menzies Institute for Medical Research, University of Tasmania, Hobart, Tasmania, Australia; 2 Medical Services Administration, Ministry of Health of the Socialist Republic of Vietnam, Hanoi, Vietnam; 3 Research Centre of Applied and Preventive Cardiovascular Medicine, University of Turku, Turku, Finland; 4 Department of Medicine, Peninsula Clinical School, Central Clinical School, Monash University, Melbourne, Victoria, Australia; University of Malaya: Universiti Malaya, MALAYSIA

## Abstract

**Background:**

The mid-life emergence of higher levels of total cholesterol (TC) for women than for men has been observed in different Western and Asian populations. The aim of this study was to investigate whether there is evidence of this in Vietnam and, if so, whether it can be explained by ageing, by body size and fatness, or by socio-demographic characteristics and behavioural factors.

**Methods:**

Participants (n = 14706, 50.9% females) aged 25–64 years were selected by multi-stage stratified cluster sampling from eight provinces each representing one of the eight geographical regions of Vietnam. Measurements were made using the World Health Organization STEPS protocols. Linear regression was used to assess the independent contributions of potential explanatory factors to mean levels of TC. Data were analysed using complex survey methods.

**Results:**

Men and women had similar mean levels of body mass index (BMI), and men had modestly higher mean levels of waist circumference (WC), in each 5-year age category. The mean TC of women increased more or less continuously across the age range but with a step-up at age 50 years to reach higher concentrations on average than those of their male counterparts. The estimated step-up was not eliminated by adjustment for anthropometric indices including BMI or WC, or by adjustment for socio-demographic characteristics or behavioural factors. The estimated step-up was least for women with the greatest weight.

**Conclusion:**

There is a marked step-up in TC at age 50 years for Vietnamese women that cannot be explained by their age, or by their body fatness or its distribution, or by their socio-demographic characteristics or behavioural factors, and which results in greater mean levels of TC for middle-aged women than for their male counterparts in Vietnam.

## Background

Raised total cholesterol is an important risk factor for ischemic heart disease and stroke, and has been estimated to be responsible for 13% of stroke deaths and 48% of ischaemic heart disease deaths [1] that together account for around 2.6 million deaths every year worldwide [2]. The global prevalence of high cholesterol, defined as total cholesterol (TC) equal to or greater than 5.0 mmol/l, was estimated to be 39% among adults aged 25 years or over in 2008 [1]. Reducing cholesterol has preventive and therapeutic benefits. It has been estimated that a 0.6 mmol/l (10%) reduction in serum cholesterol would lower the risk of ischaemic heart disease by 50% for men aged 40 years and by somewhat less for older men, with the reduction falling to 20% for men aged 70 years [3]. The American Heart Association reported the most up-to-date statistics related to heart disease that long-term exposure to even modestly elevated cholesterol levels can lead to coronary heart disease (CHD) later in life [4]. The study from 6 United States cohorts found that CHD rates were significantly elevated among young adults (18–39 years of age) who had to low-density lipoprotein (LDL) cholesterol over 100 (versus under 100) mg/dL, independently of later adults (≥40 years of age) exposures (adjusted HR, 1.64) [4]. In a 20-year follow-up study, early initiation of statin treatment among 214 children with familial hypercholesterolemia was associated with a decrease in LDL cholesterol by 32%, slowed progression of subclinical atherosclerosis, and lower cumulative incidence by 39 years of age of cardiovascular events compared with affected parents (0% versus 7% and 1% versus 26% of fatal and nonfatal cardiovascular events, respectively) [4].

Comparing men and women, mean levels of TC and LDL cholesterol tend to be higher for men than for women in early adulthood but with women catching up in mid-life and typically with the difference reversed after age 50. This pattern has been observed in different Western [5–8] and Asian populations [9–11]. Consistent with this pattern, the incidence of cardiovascular disease (CVD) among women typically lags 10 years behind that of men until the sixth or seventh decade of life when there is a marked increase in female rates [12]. Follow-up studies have suggested that LDL cholesterol rises during the transition to menopause [13–15]. This accords with cross-sectional data showing a step-up in mean levels of TC for women in their late 40s or early 50s [5, 7, 16]. Longitudinal observations of women during the menopausal transition show that changes in TC and LDL cholesterol occurred mainly during the later phases of menopause and were greatest for women who were lightest at baseline [13]. Whilst weight gain is a common occurrence for women during the menopausal years [17], weight gain appears not to be a consequence of menopause per se [18]. The results of some studies suggest that the rise in TC at these ages is independent of any changes in body size and fatness occurring at or around the same time [19, 20].

We had the opportunity to investigate sex differences in TC in a population (that of Vietnam) in which men and women are similar in weight relative to height, and predominately of the same ethnic background. Given the research evidence that the mid-life increase in TC is more pronounced for women of smaller body weight, the rise in TC may be more clearly discernible in a population of women generally smaller than their Western counterparts. Additionally, to the extent that treatment with hormone replacement therapy (HRT) by post-menopaused women can mask sex differences in TC, the lack of widespread use of exogeneous hormones in Vietnam was a benefit for this study. Furthermore, any contribution of tobacco smoking and alcohol consumption would be specific to males in this population because few of the women smoke or consume alcohol. Using data from a national survey of risk factors for non-communicable diseases (NCDs) in Vietnam, the aim of this study was to investigate whether there is evidence of higher female-than-male mean levels of TC in Vietnam and, if so, whether it can be explained by ageing, by body size and fatness, or by socio-demographic characteristics and behavioural factors.

## Methods

### Study participants and sampling

The data are from a population-based survey of risk factors for non-communicable disease in Vietnam during 2009–2010 that was designed in accordance with the STEPS survey methodology of the World Health Organization (WHO) [21]. The survey participants (n = 14,706, response proportion 64.1%) were selected by multi-stage stratified cluster sampling from eight provinces each representative of one of the eight geographical regions of Vietnam. The study was approved by the Ethics Committee of the Ministry of Health of Vietnam and the Tasmanian Health and Medical Human Research Ethics Committee. Written informed consent was obtained from participants. The details of this survey have been reported elsewhere [22].

### Measurements

The STEPS questionnaire was used to collect information on socio-demographic information and measurements of four behavioural factors (tobacco smoking, alcohol, fruit/vegetable consumption, and physical activity) [21]. The questionnaire was translated into Vietnamese and back-translated to check the accuracy of wording of each item. Our previous studies confirmed that the measurements made with the instrument—in aggregate [23], and in respect of tobacco smoking [22], physical activity [24], alcohol intake [25], and fruit and vegetable intake [26]–have validity. Face-to-face interviews were conducted with participants by trained staff of each provincial health authority.

Self-reported highest education levels were categorized as less than primary (<5 years), primary (5–8 years), junior secondary (9–11 years), senior secondary (12 years), and college/undergraduate or postgraduate (>12 years). Monthly income was answered in Vitenam Dong and transferred to USD for analysis. Smoking status were categorised as never smokers, former daily smokers, current and former non-daily smokers, and current daily smokers. For alcohol intake status, those who reported consuming at least one alcoholic beverage during the previous year were asked about their frequency of consumption (response categories <1 day/month, 1–3 days/month, 1–4 days/week, 5–6 days/week, and daily). Show cards illustrating the volume of spirits (30 ml of 40% alc/vol), wine (120 ml of 11% alc/vol) and beer (285 ml of 4.5% alc/vol) equivalent to 10 g of ethanol (a standard drink) were used to prompt reporting of the number of standard drinks usually consumed on each drinking occasion. Reported number of standard drinks were categorized as <2, 2–3, 3.1–6 and >6 standard drinks. Alcohol intake status were categorised as low (< 4 standard drinks for men or <2 standard drinks for women), hazardous (4–6 standard drinks for men or 2–4 standard drinks for women), harmful (consuming at least 6 standard drinks for men or 4 standard drinks for women). The participants were asked about the number of days they usually ate fruit, and the number of days they usually ate vegetables (excluding root plants), in a typical week and how many ‘standard serving’ sizes they usually ate of each on those days. A ‘standard serving’ size of vegetables was defined as a cup of raw vegetables, a half cup of cooked or chopped raw vegetables or half cup of vegetable juice. A ‘standard serving’ size of fruit was defined as a piece of whole of fruit, a half cup of cooked, chopped or processed fruit or half cup of fruit juice and assumed to correspond to 80 gram. Visual aids (show-cards) depicting a ‘standard serving’ size of fruit and vegetables were used to facilitate interviewing. Activity levels were calculated as total time spent on work, transport and leisure time activities of each intensity, weighted by The Global Physical Activity Questionnaire-assigned Metabolic Equivalent Task (MET) energy expenditure ratios per kilogram per hour of 4 for moderate and 8 for vigorous intensity activities. Subjects were categorised as having low (<600MET/week), moderate (>600MET/week), or high (>3000MET/week) activity levels. Measurements of behavioural factors were made and categorised according to recommendations of the WHO [21]. Physical measurements included weight (in bare feet without heavy clothing measured using NuWeigh B8271 digital scales with the precision of 0.1 kg), height (in bare feet without headwear measured using a Seca 214 stadiometer with the precision of 0.1 cm), waist circumference (WC, at the narrowest point between the lower costal border and the iliac crest measured horizontally using a constant tension tape while standing), and hip circumference (at the greatest posterior protuberance of the buttocks measured using a constant tension tape) with the participants standing. Body mass index (BMI) was calculated as weight (kg) ÷ height^2^ (m). Waist-hip-ratio (WHR) was calculated as WC (cm) ÷ hip (cm). Waist-to-height-ratio (WHtR) was calculated as WC (cm) ÷ height (cm).

After overnight fasting, TC was measured according to the standardised STEPS procedure [21] from capillary whole blood using Roche Diagnostics Accutrend Plus glucometers. Raised TC was defined as TC ≥ 5.0 mmol/L.

### Data analysis

Sampling weights were defined as the inverse probability of selection in the sample, and calculated as the product of the probability that each cluster was chosen and the probability that each person from each selected cluster was chosen. Appropriately weighted and stratified estimates of means and proportions, and of regression coefficients, were made using complex survey estimation methods provided by Stata version 15.0.

The effects of fat distribution, sociodemographic, and behavioural factors on the sex differences in TC were examined by linear regression with TC as dependent variables and body fatness including BMI, WC, WHR, WHtR, socio-demographic and behavioural factors as independent variables. TC was transformed to reduce skewness. Change-point analysis was used to identify whether a mean-shift and/or change in slope had occurred in the regression of TC on age. The change-point analyses reported in this study were designed to allow the slope of the relationships of TC with age to change if necessary from any age onwards, and/or for the relationships to shift upwards (“step-up”) or downwards (“step-down”) if necessary at any age. Candidate change-points were identified by trial-and-error and confirmed by Bayesian change-point analysis. The final models included a quadratic (increasing before decreasing) fit in age to the TC data for men, without any discrete change in slope or location, and a step-up at age 50 years in the otherwise consistently increasing linear-in-age relationship for women.

For Table 3, a linear regression model with age and a binary (0/1) term as covariates was fitted to the TC data for women. The binary term took the value 0 for women aged less than 50 years or 1 for women aged 50 years or greater. The coefficient of the binary term represents the estimated vertical displacement of the relationship and being positive, is referred to as the “step-up”. The estimated mean value of TC not including (without) and including (with) the step-up is reported, together with the step-up expressed as a percentage increase. To examine whether the step-up in cholesterol at age 50 years remained after adjustment for other factors including indices of body size and fatness, socio-demographic characteristics and behavioural attributes, a covariate for each of these factors was added in turn and the adjusted value of the step-up was used to recalculate these quantities (mean TC without step-up, mean TC with step-up, percentage increase).

## Results

Table 1 presents selected characteristics of study subjects, stratified by sex. The sample consisted of 14706 (50.9% female) participants aged 25–64 years, with generally higher participation proportions among older people. Approximately 70% of the sample lived in rural areas and most participants were of Kinh ethnicity. Men had higher average of years of schooling and were more active than women. The proportion of subjects who smoked tobacco and the proportion who consumed alcohol were much higher among men than women. There was a modest difference in mean WC between men and women but mean BMI, WHR and WHtR were similar.

**Table 1:** Characteristics* of survey participants.

	Men				Women			
	<50 years (n = 3989)		≥50years (n = 2815)		<50 years (n = 4770)		≥50years (n = 3132)	
Ethnicity								
Kinh	93.7%	(3234/3981)	95.7%	(2392/2806)	93.9%	(3942/4766)	95.5%	(2664/3123)
Non-Kinh	6.3%	(747/3981)	4.3%	(414/2806)	6.1%	(824/4766)	4.5%	(459/3123)
Residential areas								
Urban	29.3%	(1311/3989)	31.7%	(1059/2815)	31.1%	(1635/4770)	31.2%	(1188/3132)
Rural	70.7%	(2678/3989)	68.3%	(1756/2815)	68.9%	(3135/4770)	68.8%	(1944/3132)
Years of schooling	8.8	(4.0)	8.2	(4.1)	8.1	(4.1)	5.9	(3.9)
Monthly income	80.0	(107.9)	65.7	(83.1)	76.1	(82.8)	57.9	(69.3)
Smoking status								
Never smoker	31.9%	(1268/3979)	26.8%	(778/2803)	98.7%	(4683/4764)	95.5%	(2911/3122)
Ex-smoker	12.4%	(562/3979)	21.3%	(628/2803)	0.2%	(11/4764)	1.2%	(59/3122)
Daily smoker	55.7%	(2149/3979)	51.9%	(1397/2803)	0.1%	(70/4764)	3.3%	(152/3122)
Alcohol intake								
Low	56.3%	(2181/3989)	68.7%	(1967/2815)	97.2%	(4605/4770)	98.3%	(3038/3132)
Hazardous	17.5%	(717/3989)	14.2%	(387/2815)	1.9%	(120/4770)	1.2%	(69/3132)
Harmful	26.2%	(1091/3989)	17.1%	(461/2815)	0.9%	(45/4770)	0.4%	(25/3132)
Standard drinks/day	4.7	(3.6)	4.0	(3.5)	1.7	(0.0)	1.5	(0.0)
Fruit/veg intake	3.2	(2.1)	3.1	(2.0)	3.2	(2.0)	3	(1.9)
Physical activity (min)	1395.7	(1524.2)	1071	(0)	1026.4	(1359.6)	935	(1149.7)
Weight (kgs)	57.0	(9.2)	55.9	(9.3)	49.8	(7.4)	50.4	(8.5)
BMI (kg/m^2^)	21.5	(3.1)	21.4	(3.1)	21.3	(2.9)	22.0	(3.4)
WC (cms)	74.5	(8.8)	76.2	(9.2)	71.1	(8.1)	74.7	(9.6)
WHR†	0.8	(0.1)	0.9	(0.1)	0.8	(0.1)	0.9	(0.1)
WHtR‡	0.5	(0.1)	0.5	(0.1)	0.5	(0.1)	0.5	(0.1)
Cholesterol (mmol/L)	4.7	(0.7)	4.8	(0.8)	4.7	(0.7)	5.2	(0.8)
Raised cholesterol §	26.0%	(906/3794)	34.3%	(875/2700)	25.4%	(1112/4565)	53.2%	(1508/2996)

* The data reported are mean (standard deviation) or percentage (relative frequency).

^†^ Waist-to-hip ratio.

^‡^ Waist-to-height ratio.

^§^ Total cholesterol > 5 mmol/L.

Table 2 presents rank correlation coefficients to summarise the associations of TC with measures of body size and fatness, socio-demographic characteristics and behavioural factors for men and women. The anthropometric indices most strongly associated with TC were WC or measures based on girths for men, and weight or measures based on weight (particularly BMI) for women. Other factors associated with TC were age and physical activity (both men and women).

**Table 2:** Rank correlations of TC with measures of body size and fatness, socio-demographic characteristics, and behavioural factors by age group and sex.

	Men				Women			
	<50 years (n = 3989)		≥50years (n = 2815)		<50 years (n = 4770)		≥50years (n = 3132)	
Years of schooling	0.03		0.08	*	–0.03		0.03	
Monthly income	0.06	*	0.10	**	0.05	*	0.04	
Smoking status	0.08	**	0.01		0.01		0.08	
Alcohol intake status	0.05		0.02		0.05		–0.03	
Fruit/vegetable intake	–0.01		0.02		–0.01		–0.03	
Physical activity	–0.20	***	-0.16	***	–0.12	***	–0.17	***
Weight	0.21	***	0.23	***	0.21	***	0.23	***
BMI	0.26	***	0.24	***	0.25	***	0.26	***
WC	0.28	***	0.27	***	0.21	***	0.24	***
WHR†	0.24	***	0.22	***	0.15	***	0.19	***
WHtR‡	0.29	***	0.26	***	0.21	***	0.24	***

* denotes p<0.05,

** denotes p<0.01,

*** denotes p<0.001.

^†^ Waist-to-hip ratio.

^‡^ Waist-to-height ratio.

**Fig 1:**
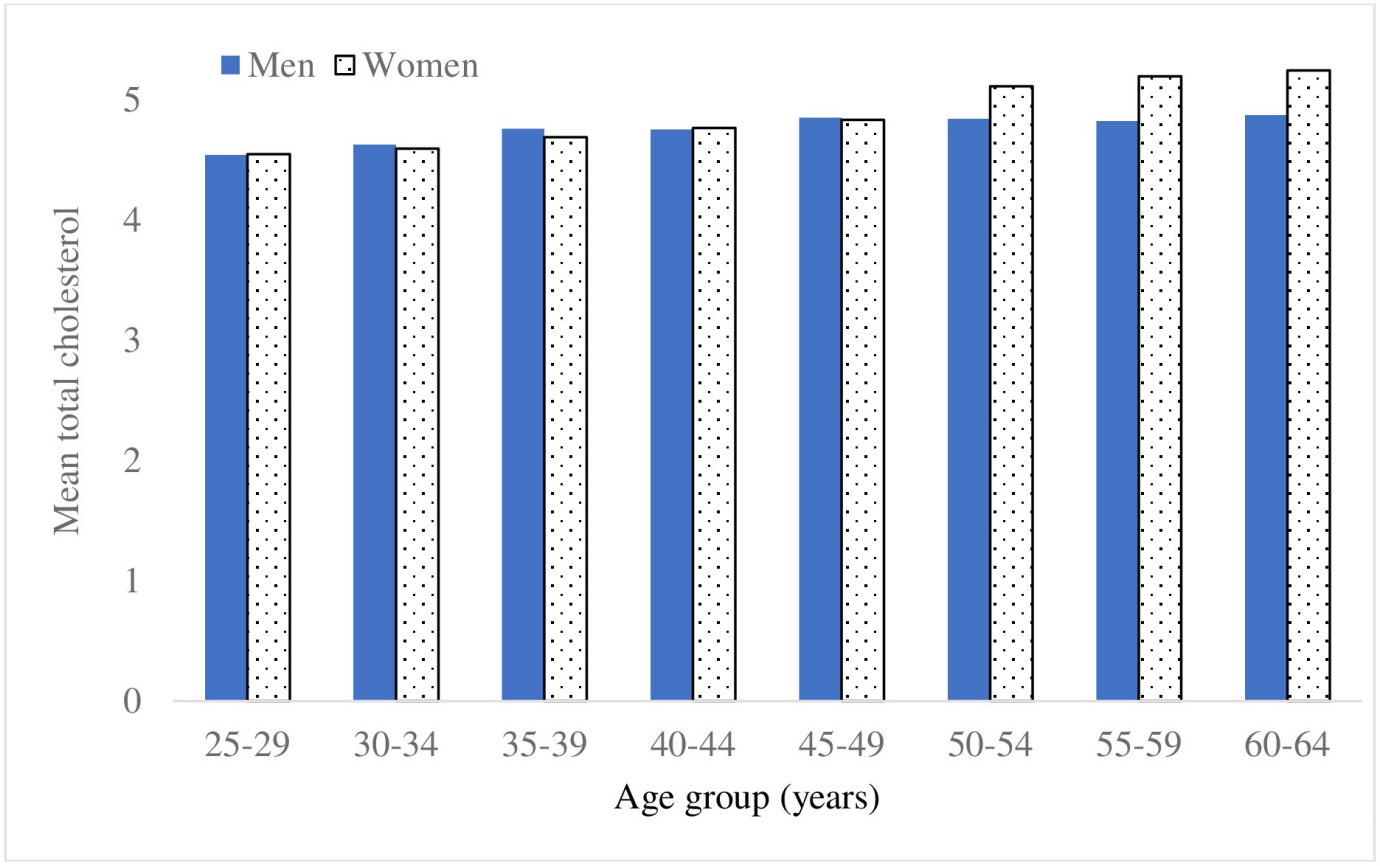
Mean of total cholesterol by age group and sex.

The sex-specific means of TC stratified by 5-year age group are depicted in Fig 1. The mean TC of men increased with age group until reaching a plateau commencing with the 45–49 years of age group. The mean TC of women increased throughout the entire age range rising from concentrations similar to those of men in young adulthood to higher values from age group 50–54 years onwards, and with a noticeable step-up occurring for the 50–54 years of age group. Commencing with that age group, the mean values of TC of middle-aged women exceeded markedly those of their male counterparts, whereas in younger age groups the mean TC levels for women were comparable to men.

**Fig 2:**
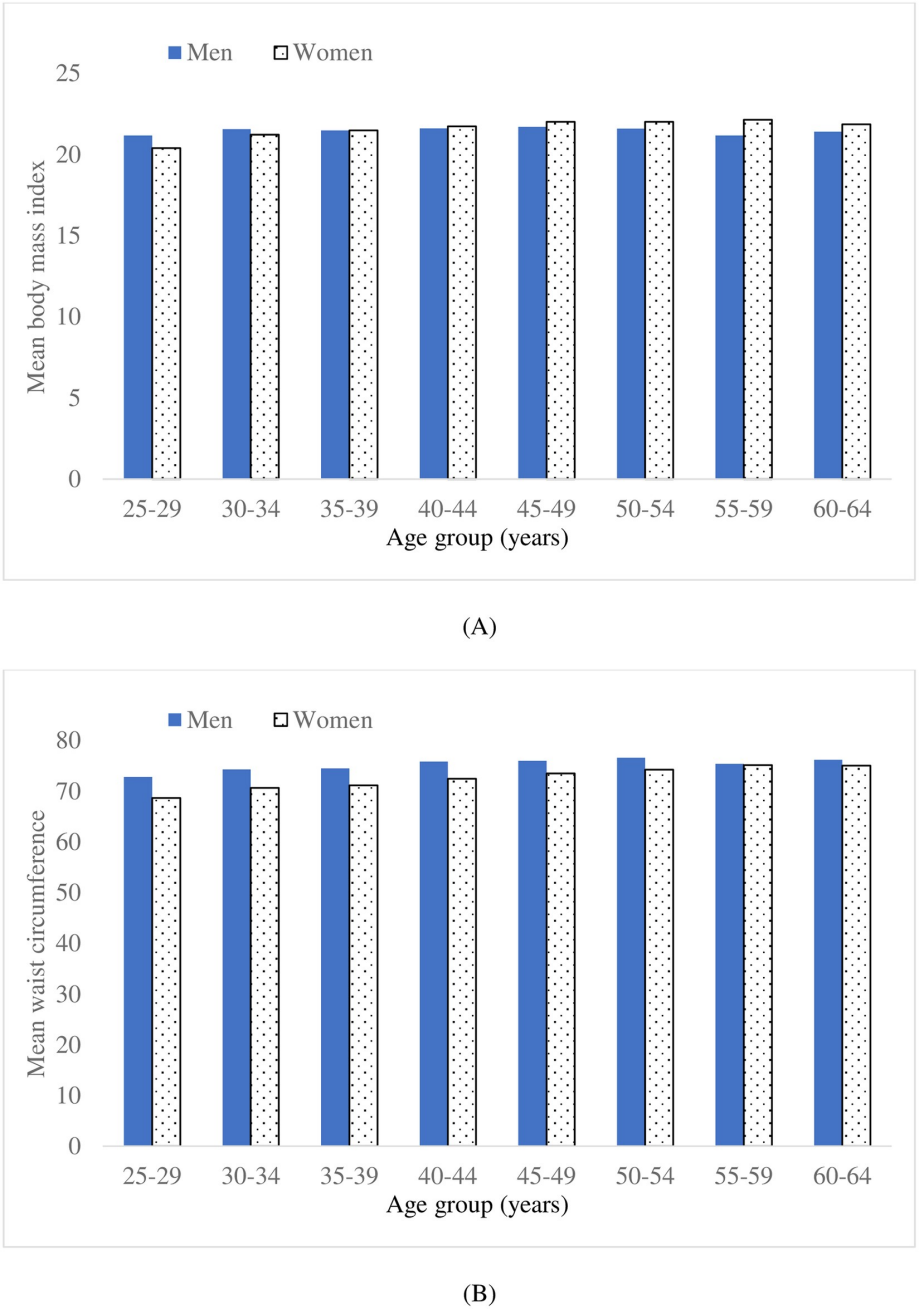
Means of body mass index (A) and waist circumference (B) by age group and sex.

The sex-specific means of two measures of body size and fatness—BMI and WC—stratified by 5-year age group are depicted in Fig 2A and 2B. The relationships with age group are mildly curvilinear in each case, with the mean values initially increasing before decreasing with advancing age group, and without visual evidence of a mean shift in the grouped data such as that in mean TC for women.

In analyses of single year of age data, a quadratic (increasing before decreasing) fit in age to the TC data was statistically significant (p = 0.002) for men. The maximum fitted mean value of TC was reached at age 56 years. For women, there was a step-up (p<0.001) of 3.5% (95% CI 1.9%, 5.1%) at age 50 years in the otherwise consistently increasing linear-in-age relationship.

In respect of the relationships of age with BMI or WC in single year of age data, quadratic (increasing before decreasing) fits in age were statistically significant for men (BMI p = 0.002, WC p<0.001) and women (BMI p<0.001, WC p<0.001). The maximum fitted mean values for men were 44 years for BMI, and 52 years for WC. The maximum fitted mean values for women were 47 years for BMI, and 57 years for WC. There were not material mean shifts for either men or women: the largest found for women were 0.7% (95% CI –1.2%, 2.6%) at age 47 years in BMI, and 0.9% (95% CI –0.5%, 2.3%) at age 48 years in WC.

In consequence, adjusting for BMI, WC or other anthropometric indices did not eliminate the estimated step-up in TC for women at age 50. Instead, Table 3 shows that the estimate was increased by adjustment for weight or BMI. That increase on adjustment requires the step-up to be greatest, on average, for women of lesser weight or BMI. Adjusting instead or adjusting additionally for indices based on girth produced only minor reductions in the estimated step-up. Adjustment for socio-demographic characteristics and behavioural factors for women that were associated with TC levels had limited impact also (see Table 3).

**Table 3:** Estimates of the step-up in the mean value of TC occurring at around 50 years of age for women in Vietnam.

	Estimate of age at step-up	TC without step-up		TC with step-up		Percentage increase	
		Mean	(95% CI)	Mean	(95% CI)	Step-up	(95% CI)
Unadjusted	50	4.73	(4.68, 4.77)	4.89	(4.85, 4.93)	3.5%	(1.9%, 5.1%)
Adjusted for							
Weight	50	4.70	(4.65, 4.75)	4.90	(4.86, 4.94)	4.2%	(2.6%, 5.8%)
BMI	50	4.70	(4.65, 4.75)	4.88	(4.84, 4.93)	4.0%	(2.4%, 5.6%)
WC	50	4.72	(4.67, 4.77)	4.88	(4.84, 4.93)	3.5%	(1.9%, 5.1%)
WHR	50	4.73	(4.68, 4.78)	4.88	(4.84, 4.93)	3.3%	(1.6%, 4.9%)
WtHR	50	4.72	(4.67, 4.77)	4.87	(4.83, 4.92)	3.3%	(1.7%, 4.9%)
BMI and WC	50	4.70	(4.65, 4.75)	4.88	(4.84, 4.92)	3.9%	(2.3%, 5.5%)
BMI and WHR	50	4.70	(4.65, 4.75)	4.88	(4.84, 4.92)	3.8%	(2.2%, 5.4%)
BMI and WtHR	50	4.70	(4.65, 4.75)	4.88	(4.84, 4.92)	3.8%	(2.2%, 5.4%)
BMI and years of schooling	50	4.70	(4.65, 4.75)	4.87	(4.83, 4.92)	3.7%	(2.1%, 5.4%)
BMI and monthly income	50	4.70	(4.65, 4.75)	4.88	(4.83, 4.93)	3.9%	(2.1%, 5.7%)
BMI and smoking	50	4.69	(4.48, 4.89)	5.00	(4.79, 5.21)	6.7%	(-1%, 14.4%)
BMI and alcohol	50	4.62	(4.50, 4.73)	4.85	(4.74, 4.97)	5.1%	(1.2%, 9.0%)
BMI and fruit/vegetable intake	50	4.70	(4.65, 4.74)	4.88	(4.83, 4.92)	3.9%	(2.2%, 5.5%)
BMI and physical activity	50	4.76	(4.71, 4.82)	4.94	(4.89, 4.99)	3.7%	(2.0%, 5.4%)
Stratified by weight category*							
Lowest third of weight for age	48	4.53	(4.44, 4.61)	4.70	(4.62, 4.78)	3.9%	(0.6%, 7.1%)
Middle third of weight for age	50	4.62	(4.54, 4.71)	4.93	(4.86, 5.01)	6.7%	(3.8%, 9.7%)
Highest third of weight for age	50	4.91	(4.82, 5)	5.05	(4.98, 5.12)	2.9%	(0.1%, 5.6%)

* For women of age 50 years, the weight tertiles were 46.7, 53.6 and 70.7 kgs, and the thirds were 34.0–46.7 kgs, 46.8–53.6 kgs and 53.7–70.7 kgs.

^†^ Weight-to-(height)^2^ ratio.

^‡^ Waist-to-hip ratio.

^§^ Waist-to-height ratio.

Also shown in Table 3 are estimates of the step-up within strata of weight for age. In this sample, the greatest step-up was that estimated for women in the middle third of weight for age. The estimated step-up was least for women in the highest third, and intermediate for women in the lowest third. At age 50 years, the range of weights for women in this sample were 34.0–46.7 kgs (lowest third), 46.8–53.6 kgs (middle third) and 53.7–70.7 kgs (highest third). The results (not shown) for stratifications by BMI or girths resembled very closely those for stratification by weight.

## Discussion

The key finding of this cross-sectional study was that the mean TC of women increased more of less continuously across the age range but with a step-up at age 50 years to reach higher concentrations than those of their male counterparts in a reversal of the male-female difference at lesser adult ages. The estimated step-up was not eliminated by adjustment for anthropometric indices including BMI or WC, or for socio-demographic characteristics or behavioural factors. The estimated step-up was least for women with greatest weight or greatest weight relative to height or WC.

Higher concentrations of TC among young adult men than among young adult women, and the reversal of the sex difference at mid-life that we observed, have been described previously in published reports of studies conducted in Netherlands [7] and England [14]. The reversal can be identified in summary data from cross-sectional surveys conducted in Australia [5]. In Asian populations, the same has been observed in Korea [16, 27] China [9], and India [11].

There is general consensus that the increases in TC across the young to middle-aged adult lifespan are related to increasing body size and fatness. The distribution of body fat may be an important determinant of sex differences in lipids, but possibly more so for triglycerides and HDL cholesterol than for TC [20, 28]. In our cross-sectional data, the women reached higher peak values of BMI and similar peak values of WC in their mid-50s, around 3 to 5 years of age later than the men. For this reason, it might be expected that the higher male-than-female mean values of TC found among younger subjects would be reversed among those subjects who had reached mid-life. On the other hand, our cross-sectional data on body size and fat distribution provided no reason to expect the step-up in TC for women that was found at age 50 years. In consequence, adjusting for anthropometric indices of body size and fatness attenuate the estimate of the step-up suggesting it was not due to increases in that factor.

Ageing, menopause, and increased risk of CVD for women occur concurrently [15]. Lipid profiles change around the time of menopause, in part related to chronologic aging and in part related to the menopausal transition itself [15]. Changes in TC during the menopausal transition for middle-aged women have been observed in both cross-sectional and longitudinal studies. A cross-sectional analysis of 3,312 UK midlife women free from hormonal treatment demonstrated women who were post-menopausal, compared with those who were pre-menopausal, had higher concentrations of multiple lipoprotein lipids, particularly LDL-cholesterol, and increased concentrations of fatty acids and several non-lipid measures [14].

The menopausal transition has been linked with an accelerated increase in TC of women in longitudinal studies conducted in the United States [13, 29], Australia [30], and China [10]. The estimated step-up in this study occurred at an age by which many or most women would have completed or nearly completed the menopausal transition. The average age of menopause in Vietnam has been reported to be 48 or 49 years [31], and this coincides remarkably closely with the pronounced increases in mean levels of TC at age 50 years we observed. Biologically, estrogens are a group of sex hormones that promote the development and maintenance of female characteristics in the human body [32]. Estrogen can reduce triglycerides synthesis, increase liver uptake of LDL-cholesterol and secretion of cholic acid, accelerate cholesterol removal in vivo, and thereby reduce the serum triglycerides, TC and LDL-cholesterol levels [33, 34]. Reduced estrogen levels at menopause dampen the metabolic activity of macrophages, and thereby contribute to lipid accumulation.

A consideration in this study was whether weight modifies the relationship between TC and estrogen levels during the menopausal transition. In the Study of Women’s Health Across the Nation (SWAN), a large prospective study conducted in 7 sites in the USA, the heaviest women had the smallest increases in TC while the lightest women experienced the greatest increases in TC during the menopausal transition [13]. In this population sample also, the estimated step-up in TC was markedly lower for women of greatest weight or greatest weight relative to height, or greatest WC. The lightest Vietnamese women did not have the greatest step-up, however. Those women were much lighter in weight than any in the SWAN, and it may be that the SWAN finding does not extend to the lower end of the range of body weights that exists for women in the Vietnamese population.

Sociodemographic characteristics and lifestyle factors may have an effect on lipid profiles [35, 36], and associations of these factors with TC have been observed in some studies [37, 38]. Nevertheless, these factors did not explain the step-up in TC among middle-age Vietnamese women. This is consistent with the findings of longitudinal studies of mainly Western women in the United States [13] and Melbourne [30].

This study has several strengths. The data were collected from a nationally-representative survey of the Vietnamese population. The large sample was stratified by sex and rural/urban location, and the availability of data on range of lifestyle risk factors for non-communicable disease made it possible to take account of potential confounding factors. All measurements were made by trained staff in accordance with standardised protocols designed by the WHO. All participants fasted in preparation for the test of TC. That the Vietnamese population is relatively slim in comparison to Western populations, and naïve in respect of HRT, arguably provided greater opportunity to observe increases in the TC concentrations of the middle-aged women in this study. That few of the women smoked or drank alcohol might have been an advantage also.

However, this study has some limitations that need to be taken into account. First, whilst the response proportion was high given the demands of participation in the study, the possibility of non-participation bias cannot be discounted. Second, some environmental factors such as years of smoking, alcohol consumption, fruit/vegetable serves, and physical activity were self-reported. Nevertheless, we used the STEPS standardised questionnaires administered in accordance with WHO protocols in an attempt to ensure consistency in measurement, and in our hands, the self-reported data had some evidence of construct validity [23–26]. Third, study participants contributed a single blood sample for cholesterol assessment, and hence intraindividual variation in cholesterol level could not be assessed or taken into account. Moreover, participants were excluded if they reported taking medication to lower TC. Fourth, information was not collected on the menopausal status of our female participants. Those aged 50 years would have been in a mixture of stages of menopause, and this is likely to have obscured partially the true step-up that occurred. In addition, we did not have information on use of HRT, though it is thought to be limited in Vietnam and confined to women of higher wealth. Fifth, we did not have detailed measurements of some important risk factors for NCD including diet. Failing to adjust for such factors may have influenced the findings. Sixth, this paper does not assess the influence of comorbidities related to blood lipid concentrations, because there was no need to. Elsewhere we have described the inter-dependence of raised blood pressure and elevated blood glucose [39] that should prevent hypertension and hyperglycaemia being modelled in isolation of each other. Seventh, general adiposity and central adiposity that BMI and waist circumference are surrogate markers at best of whatever it is about body size and fatness that confers risk of hypercholesteremia. Finally, we cannot discount cohort effects in this study given that these are cross-sectional data.

In conclusion, there is a marked step-up in TC at age 50 years for Vietnamese women that cannot be explained by their age, or by their body fatness or its distribution, or by their socio-demographic characteristics or behavioural factors, and which results in greater mean levels of TC for middle-aged women than for their male counterparts in Vietnam. Cholesterol reduction is effective in reducing morbidity and mortality from CVD, and there may be a case for the Government of the Socialist Republic of Vietnam to invest in HRT. Standard clinic guidelines on HRT in Vietnam have not been developed or issued. The gender and age differences in TC levels found in this study suggest that middle-aged Vietnamese women should be the prioritized target for better control of dyslipidaemia and early prevention of cardiovascular disease.

## Supporting information

S1 Appendix Study sampling method. (DOCX)

S2 Appendix Stages of sampling (PPS = probability proportional to size of population). (DOCX)
