# Supplementary material for: Sex differences in total cholesterol of Vietnamese adults
Source: PLoS One. 2021 Aug 20;16(8):e0256589. doi: 10.1371/journal.pone.0256589 (PMC8378708; doi:10.1371/journal.pone.0256589)
Supplement: S1 Appendix — (DOCX) [file pone.0256589.s001.docx]

S1 Appendix: Study sampling method

The survey participants were 25 to 64-year-old Vietnamese residents (n=14,706, response proportion 64.1% of the 22,940 eligible subjects) selected by multi-stage stratified cluster sampling from eight provinces (Thai Nguyen, Hoa Binh, Ha Noi, Hue, Binh Dinh, Dak Lak, Ho Chi Minh City and Can Tho) each representative of one of the eight geographical regions of Vietnam. Sampling procedures and measurements were made in accordance with the STEPS methodology [21]. The two-stage sampling procedure involved selecting 20 clusters (communes, towns, and city wards) from each of the eight geographically representative provinces with probabilities proportional to population size from four strata defined by urban-rural location and rich-poor classification. For each selected cluster, the provincial health authority prepared a comprehensive listing of 25-64-year-old residents. From those lists, adequate numbers of persons per cluster were selected by age- and sex-stratified random sampling to provide 25 persons in each age group (25−34 years, 35−44 years, 45−54 years, 55−64 years) and with approximately equal members of men and women. Clinics were conducted in the local health station of each participant’s area of residence. Interviewers were local medical personnel who were trained in the implementation of the WHO STEPS methodology. Training of field staff was conducted pre-survey at training centres in Ha Noi, Hue and Ho Chi Minh City, and on-site at regular intervals by local, national and international supervisors. The eligible persons were invited to attend a clinic on a specific date, each clinic commencing in the early morning because overnight fasting was required, and questionnaire data were obtained by face-to-face interview at the survey clinics. Measurements were made, and questionnaires were administered, by trained staff of each provincial health authority. They underwent intensive training and supervision provided by the Menzies Institute for Medical Research, Australia. A pilot study was conducted to test survey instruments and procedures before actual data collection. All measurements were performed in accordance with the STEPS protocols.
