# Supplementary material for: Sex differences in total cholesterol of Vietnamese adults
Source: PLoS One. 2021 Aug 20;16(8):e0256589. doi: 10.1371/journal.pone.0256589 (PMC8378708; doi:10.1371/journal.pone.0256589)
Supplement: S2 Appendix — (DOCX) [file pone.0256589.s002.docx]

S2 Appendix: Stages of sampling (PPS = Probability Proportional to Size of population)
