# Supplementary material for: Sex differences in total cholesterol of Vietnamese adults
Source: PLoS One. 2021 Aug 20;16(8):e0256589. doi: 10.1371/journal.pone.0256589 (PMC8378708; doi:10.1371/journal.pone.0256589)
Supplement: Peer review history

# Peer review: Decision Letter 0

**DOI:** 10.1371/journal.pone.0256589.r001

**Authors:** Lee-Ling Lim

12 May 2021

PONE-D-20-37824

Factors that influence sex differences in total cholesterol of Vietnamese adults

PLOS ONE

Dear Dr. Blizzard,

Thank you for submitting your manuscript to PLOS ONE. After careful consideration, we feel that it has merit but does not fully meet PLOS ONE’s publication criteria as it currently stands. Therefore, we invite you to submit a revised version of the manuscript that addresses the points raised during the review process.

Please submit your revised manuscript by Jun 24 2021 11:59PM. If you will need more time than this to complete your revisions, please reply to this message or contact the journal office at plosone@plos.org. When you're ready to submit your revision, log on to https://www.editorialmanager.com/pone/ and select the 'Submissions Needing Revision' folder to locate your manuscript file.

Please include the following items when submitting your revised manuscript:

- A rebuttal letter that responds to each point raised by the academic editor and reviewer(s). You should upload this letter as a separate file labeled 'Response to Reviewers'.
- A marked-up copy of your manuscript that highlights changes made to the original version. You should upload this as a separate file labeled 'Revised Manuscript with Track Changes'.
- An unmarked version of your revised paper without tracked changes. You should upload this as a separate file labeled 'Manuscript'.

If you would like to make changes to your financial disclosure, please include your updated statement in your cover letter. Guidelines for resubmitting your figure files are available below the reviewer comments at the end of this letter.

If applicable, we recommend that you deposit your laboratory protocols in protocols.io to enhance the reproducibility of your results. Protocols.io assigns your protocol its own identifier (DOI) so that it can be cited independently in the future. For instructions see: http://journals.plos.org/plosone/s/submission-guidelines#loc-laboratory-protocols. Additionally, PLOS ONE offers an option for publishing peer-reviewed Lab Protocol articles, which describe protocols hosted on protocols.io. Read more information on sharing protocols at https://plos.org/protocols?utm_medium=editorial-email&utm_source=authorletters&utm_campaign=protocols.

We look forward to receiving your revised manuscript.

Kind regards,

Lee-Ling Lim

Academic Editor

PLOS ONE

Additional Editor Comments (if provided):

1) Please explain why only total cholesterol was examined.

2) Additional analyses on LDL cholesterol and/or non-HDL cholesterol will be helpful. The Introduction section also discussed about the implications of LDL cholesterol.

3) Include the sampling method as an Appendix.

4) Include a study flow diagram about patient selection.

Journal Requirements:

When submitting your revision, we need you to address these additional requirements.

1) Please ensure that your manuscript meets PLOS ONE's style requirements, including those for file naming. The PLOS ONE style templates can be found at

https://journals.plos.org/plosone/s/file?id=wjVg/PLOSOne_formatting_sample_main_body.pdf and

https://journals.plos.org/plosone/s/file?id=ba62/PLOSOne_formatting_sample_title_authors_affiliations.pdf

2) Please note that according to our submission guidelines (http://journals.plos.org/plosone/s/submission-guidelines), outmoded terms and potentially stigmatizing labels should be changed to more current, acceptable terminology. For example: “Caucasian” should be changed to “white” or “of [Western] European descent” (as appropriate).

3) Thank you for stating the following financial disclosure:

 [Yes. This work was supported by The Atlantic Philanthropies Inc, United States (grant

number G0015338). NTTT is supported by a Tasmania Graduate Research

Scholarship; LB was supported by a National Health and Medical Research Council

Career Development Fellowship. SLG is supported by a National Heart Foundation of

Australia Future Leader Fellowship (FLF100446). MC is supported by a National

Health and Medical Research Council Boosting Dementia Leadership Research

Fellowship (APP1135761).].          

Please state what role the funders took in the study.  If the funders had no role, please state: "The funders had no role in study design, data collection and analysis, decision to publish, or preparation of the manuscript."

If this statement is not correct you must amend it as needed.

Please include this amended Role of Funder statement in your cover letter; we will change the online submission form on your behalf.

4) Thank you for stating the following in the Competing Interests section:

[The authors have declared that no competing interests exist.]. 

We note that you received funding from a commercial source: Atlantic Philanthropies Inc,

Please provide an amended Competing Interests Statement that explicitly states this commercial funder, along with any other relevant declarations relating to employment, consultancy, patents, products in development, marketed products, etc.

Within this Competing Interests Statement, please confirm that this does not alter your adherence to all PLOS ONE policies on sharing data and materials by including the following statement: "This does not alter our adherence to PLOS ONE policies on sharing data and materials.” (as detailed online in our guide for authors http://journals.plos.org/plosone/s/competing-interests).  If there are restrictions on sharing of data and/or materials, please state these. Please note that we cannot proceed with consideration of your article until this information has been declared.

Please include your amended Competing Interests Statement within your cover letter. We will change the online submission form on your behalf.

Please know it is PLOS ONE policy for corresponding authors to declare, on behalf of all authors, all potential competing interests for the purposes of transparency. PLOS defines a competing interest as anything that interferes with, or could reasonably be perceived as interfering with, the full and objective presentation, peer review, editorial decision-making, or publication of research or non-research articles submitted to one of the journals. Competing interests can be financial or non-financial, professional, or personal. Competing interests can arise in relationship to an organization or another person. Please follow this link to our website for more details on competing interests: http://journals.plos.org/plosone/s/competing-interests

[Note: HTML markup is below. Please do not edit.]

Reviewers' comments:

Reviewer's Responses to Questions

**Comments to the Author**

1. Is the manuscript technically sound, and do the data support the conclusions?

The manuscript must describe a technically sound piece of scientific research with data that supports the conclusions. Experiments must have been conducted rigorously, with appropriate controls, replication, and sample sizes. The conclusions must be drawn appropriately based on the data presented. 

Reviewer #1: No

Reviewer #2: Yes

**********

2. Has the statistical analysis been performed appropriately and rigorously? 

Reviewer #1: Yes

Reviewer #2: Yes

**********

3. Have the authors made all data underlying the findings in their manuscript fully available?

The PLOS Data policy requires authors to make all data underlying the findings described in their manuscript fully available without restriction, with rare exception (please refer to the Data Availability Statement in the manuscript PDF file). The data should be provided as part of the manuscript or its supporting information, or deposited to a public repository. For example, in addition to summary statistics, the data points behind means, medians and variance measures should be available. If there are restrictions on publicly sharing data—e.g. participant privacy or use of data from a third party—those must be specified.

Reviewer #1: No

Reviewer #2: Yes

**********

4. Is the manuscript presented in an intelligible fashion and written in standard English?

PLOS ONE does not copyedit accepted manuscripts, so the language in submitted articles must be clear, correct, and unambiguous. Any typographical or grammatical errors should be corrected at revision, so please note any specific errors here.

Reviewer #1: Yes

Reviewer #2: Yes

**********

5. Review Comments to the Author

Please use the space provided to explain your answers to the questions above. You may also include additional comments for the author, including concerns about dual publication, research ethics, or publication ethics. (Please upload your review as an attachment if it exceeds 20,000 characters)

Reviewer #1: This paper tried to utilize the existing cohort data in 2009-2010 to demonstrate whether any differences in lipid profiles pattern between sexes. However, several pitfalls and some improvements should be acknowledged and maybe more suitable in other journals as followings ...

1. The title did not congruent with the content of the study.

2. Review literature in both background and discussions should be more updated with latest publications. Also, mechanistic studies should be more explained in the discussions.

3. Total cholesterol from capillary method might not be reflected for LDL which involves in the process of atherosclerosis.

4. Figures should be thoroughly demonstrated in the upper range of age group.

5. Table should be compared between age group of under/over 50 years in each sex.

6. Weight circumference should be acknowledged for the pitfall as a surrogate marker of visceral fat.

Reviewer #2: Dear authors:

I congratulate you on your work. Studies with such a significant sample size and multi-center are not always found. The introduction provides a useful review of the research field and the significance of lipid disorders (TC) as a public health concern. The objectives of the manuscript are clearly enumerated. There is strong concordance between the objectives and the methods used. The ethical issues were considered. The data set is complete, and the results are presented in detail. The discussion correlates well with the presented data and takes the published literature into account

Although I consider that the work has the potential to be published, there are some issues that should deserve your attention:

Introduction

Your choice was to reference many studies that has more than 10 years. In my opinion there is no need of use 'old' studies, because there are several recent studies that allows to reformulate the introduction and the discussion and keep the oldest references associated with the data collection instruments.

Methods

Line 123-125: please clarify how to select participants, include and exclude criteria.

Line 130 - 132: more information should be provided such as provide the categories of educational level (year of schooling); monthly income (Vietnam Dong or USD?); smoking status, alcohol intake status, fruit/vegetable intake (how to measure?). How to evaluate physical activity?

Line 132-133: “The questionnaire was translated into Vietnamese and back-translated to check the accuracy of wording of each item”. The manuscript must be revised to provide a hint at the validity of the so-constructed research tool.

Discussion:

One of the limitations is that the author does not discuss comorbidities related to blood lipid concentrations (hypertension, diabetes, dyslipidemia…).

Another limitation is related to not including blood lipid-lowering medicine.

Conclusions

You also need to state the implications of the study findings for future research studies, clinical leadership, and policy in the conclusion section

References

The references included are relevant for the subject under study, but only show 8/47(17%) references from the last 5 years.

**********

6. PLOS authors have the option to publish the peer review history of their article (what does this mean?). If published, this will include your full peer review and any attached files.

If you choose “no”, your identity will remain anonymous but your review may still be made public.

**Do you want your identity to be public for this peer review?** For information about this choice, including consent withdrawal, please see our Privacy Policy.

Reviewer #1: No

Reviewer #2: **Yes: **Vu Thi Thanh Huyen, Hanoi Medical University

[NOTE: If reviewer comments were submitted as an attachment file, they will be attached to this email and accessible via the submission site. Please log into your account, locate the manuscript record, and check for the action link "View Attachments". If this link does not appear, there are no attachment files.]

While revising your submission, please upload your figure files to the Preflight Analysis and Conversion Engine (PACE) digital diagnostic tool, https://pacev2.apexcovantage.com/. PACE helps ensure that figures meet PLOS requirements. To use PACE, you must first register as a user. Registration is free. Then, login and navigate to the UPLOAD tab, where you will find detailed instructions on how to use the tool. If you encounter any issues or have any questions when using PACE, please email PLOS at figures@plos.org. Please note that Supporting Information files do not need this step.

# Author comment: Author response to Decision Letter 0

**DOI:** 10.1371/journal.pone.0256589.r002

13 Jul 2021

Editor

1) Please explain why only total cholesterol was examined.

 After overnight fasting, TC was measured from capillary whole blood using Roche Diagnostics Accutrend Plus glucometers. This method is appropriate for large-scale field work (n = 14,706 in the case of our survey), and was done in accordance with the standardised STEPS protocols [21].

2) Additional analyses on LDL cholesterol and/or non-HDL cholesterol will be helpful. The Introduction section also discussed about the implications of LDL cholesterol.

We do not have the data on LDL and non-HDL cholesterol. We acknowledged this as one of limitations of the study. In the absence of measurements of LDL cholesterol, the implications of LDL cholesterol were not mentioned in the Introduction. 

3) Include the sampling method as an Appendix.

We have added the sampling method as Appendix 1 of the study. The added text is:

Appendix 1: Study sampling method 

The survey participants were 25 to 64-year-old Vietnamese residents (n=14,706, response proportion 64.1% of the 22,940 eligible subjects) selected by multi-stage stratified cluster sampling from eight provinces (Thai Nguyen, Hoa Binh, Ha Noi, Hue, Binh Dinh, Dak Lak, Ho Chi Minh City and Can Tho) each representative of one of the eight geographical regions of Vietnam. Sampling procedures and measurements were made in accordance with the STEPS methodology [21] The two-stage sampling procedure involved selecting 20 clusters (communes, towns, and city wards) from each of the eight geographically representative provinces with probabilities proportional to population size from four strata defined by urban-rural location and rich-poor classification. For each selected cluster, the provincial health authority prepared a comprehensive listing of 25-64-year-old residents. From those lists, adequate numbers of persons per cluster were selected by age- and sex-stratified random sampling to provide 25 persons in each age group (25−34 years, 35−44 years, 45−54 years, 55−64 years) and with approximately equal members of men and women. Clinics were conducted in the local health station of each participant’s area of residence. Interviewers were local medical personnel who were trained in the implementation of the WHO STEPS methodology. Training of field staff was conducted pre-survey at training centres in Ha Noi, Hue and Ho Chi Minh City, and on-site at regular intervals by local, national and international supervisors. The eligible persons were invited to attend a clinic on a specific date, each clinic commencing in the early morning because overnight fasting was required, and questionnaire data were obtained by face-to-face interview at the survey clinics. Measurements were made, and questionnaires were administered, by trained staff of each provincial health authority. They underwent intensive training and supervision provided by the Menzies Institute for Medical Research, Australia. A pilot study was conducted to test survey instruments and procedures before actual data collection. All measurements were performed in accordance with the STEPS protocols. 

4) Include a study flow diagram about patient selection.

We have added the flow diagram about patient selection as Appendix 2 of the study. The added diagram is:

Appendix 2: Stages of sampling (PPS = Probability Proportional to Size of population)

Reviewer #1

1. The title did not congruent with the content of the study.

We have changed the title to “Sex differences in total cholesterol of Vietnamese adults”.

2. Review literature in both background and discussions should be more updated with latest publications. Also, mechanistic studies should be more explained in the discussions.

Literature has been revised and updated with latest publications. 

3. Total cholesterol from capillary method might not be reflected for LDL which involves in the process of atherosclerosis.

Yes, we acknowledged this as one of limitations of this study. 

4. Figures should be thoroughly demonstrated in the upper range of age group.

The figures have been drawn to improve their clarity. 

Figure 1. Mean of total cholesterol by age group and sex

(A)

(B)

Figure 2. Mean of body mass index (A) and waist circumference (B) by age group and sex

5. Table should be compared between age group of under/over 50 years in each sex.

Table 1: Characteristics* of survey participants

 Men Women 

 <50 years

(n=3989) >50years

(n=2815) <50 years

(n=4770) >50years

(n=3132)

Ethnicity 

 Kinh 93.7% (3234/3981) 95.7% (2392/2806) 93.9% (3942/4766) 95.5% (2664/3123)

 Non-Kinh 6.3% (747/3981) 4.3% (414/2806) 6.1% (824/4766) 4.5% (459/3123)

Residential areas 

 Urban 29.3% (1311/3989) 31.7% (1059/2815) 31.1% (1635/4770) 31.2% (1188/3132)

 Rural 70.7% (2678/3989) 68.3% (1756/2815) 68.9% (3135/4770) 68.8% (1944/3132)

Years of schooling 8.8 (4.0) 8.2 (4.1) 8.1 (4.1) 5.9 (3.9)

Monthly income 80.0 (107.9) 65.7 (83.1) 76.1 (82.8) 57.9 (69.3)

Smoking status 

 Never smoker 31.9% (1268/3979) 26.8% (778/2803) 98.7% (4683/4764) 95.5% (2911/3122)

 Ex-smoker 12.4% (562/3979) 21.3% (628/2803) 0.2% (11/4764) 1.2% (59/3122)

 Daily smoker 55.7% (2149/3979) 51.9% (1397/2803) 0.1% (70/4764) 3.3% (152/3122)

Alcohol intake 

 Low 56.3% (2181/3989) 68.7% (1967/2815) 97.2% (4605/4770) 98.3% (3038/3132)

 Hazardous 17.5% (717/3989) 14.2% (387/2815) 1.9% (120/4770) 1.2% (69/3132)

 Harmful 26.2% (1091/3989) 17.1% (461/2815) 0.9% (45/4770) 0.4% (25/3132)

Standard drinks/day 4.7 (3.6) 4.0 (3.5) 1.7 (0.0) 1.5 (0.0)

Fruit/veg intake 3.2 (2.1) 3.1 (2.0) 3.2 (2.0) 3 (1.9)

Physical activity (min) 1395.7 (1524.2) 1071 (0) 1026.4 (1359.6) 935 (1149.7)

Weight (kgs) 57.0 (9.2) 55.9 (9.3) 49.8 (7.4) 50.4 (8.5)

BMI (kg/m²) 21.5 (3.1) 21.4 (3.1) 21.3 (2.9) 22.0 (3.4)

WC (cms) 74.5 (8.8) 76.2 (9.2) 71.1 (8.1) 74.7 (9.6)

WHR† 0.8 (0.1) 0.9 (0.1) 0.8 (0.1) 0.9 (0.1)

WHtR‡ 0.5 (0.1) 0.5 (0.1) 0.5 (0.1) 0.5 (0.1)

Cholesterol (mmol/L) 4.7 (0.7) 4.8 (0.8) 4.7 (0.7) 5.2 (0.8)

Raised cholesterol §

26.0% (906/3794) 34.3% (875/2700) 25.4% (1112/4565) 53.2% (1508/2996)

* The data reported are mean (standard deviation) or percentage (relative frequency). 

† Waist-to-hip ratio

‡ Waist-to-height ratio

§ Total cholesterol > 5 mmol/L.

Table 2: Rank correlations of TC with measures of body size and fatness, socio-demographic characteristics, and behavioural factors by age group and sex

 Men Women 

 <50 years

(n=3989) >50years

(n=2815) <50 years

(n=4770) >50years

(n=3132)

Years of schooling 0.03 0.08 * –0.03 0.03 

Monthly income 0.06 * 0.10 ** 0.05 * 0.04 

Smoking status 0.08 ** 0.01 0.01 0.08 

Alcohol intake status 0.05 0.02 0.05 –0.03 

Fruit/vegetable intake –0.01 0.02 –0.01 –0.03 

Physical activity –0.20 *** -0.16 *** –0.12 *** –0.17 ***

Weight 0.21 *** 0.23 *** 0.21 *** 0.23 ***

BMI 0.26 *** 0.24 *** 0.25 *** 0.26 ***

WC 0.28 *** 0.27 *** 0.21 *** 0.24 ***

WHR† 0.24 *** 0.22 *** 0.15 *** 0.19 ***

WHtR‡ 0.29 *** 0.26 *** 0.21 *** 0.24 ***

* denotes p<0.05, ** denotes p<0.01, *** denotes p<0.001

† Waist-to-hip ratio

‡ Waist-to-height ratio.

6. Weight circumference should be acknowledged for the pitfall as a surrogate marker of visceral fat.

We have added a note to this effect as a limitation. The added text is:

General adiposity and central adiposity that BMI and waist circumference are surrogate markers at best of whatever it is about body size and fatness that confers risk of hypercholesteremia. 

Reviewer #2: 

1. Introduction

Your choice was to reference many studies that has more than 10 years. In my opinion there is no need of use 'old' studies, because there are several recent studies that allows to reformulate the introduction and the discussion and keep the oldest references associated with the data collection instruments.

The literature review component of the Introduction has been updated.

2. Methods

Line 123-125: please clarify how to select participants, include and exclude criteria.

To clarify, we have added a description of the sampling method as Appendix 1 of the study

Line 130 - 132: more information should be provided such as provide the categories of educational level (year of schooling); monthly income (Vietnam Dong or USD?); smoking status, alcohol intake status, fruit/vegetable intake (how to measure?). How to evaluate physical activity?

We have revised the text of the Measurements section. The amended text (lines 135-163) is:

Self-reported highest education levels were categorized as less than primary (<5 years), primary (5–8 years), junior secondary (9–11 years), senior secondary (12 years), and college/undergraduate or postgraduate (>12 years). Monthly income was answered in Vitenam Dong and transferred to USD for analysis. Smoking status were categorised as never smokers, former daily smokers, current and former non-daily smokers, and current daily smokers. For alcohol intake status, those who reported consuming at least one alcoholic beverage during the previous year were asked about their frequency of consumption (response categories <1 day/month, 1–3 days/month, 1–4 days/week, 5–6 days/week, and daily. Show cards illustrating the volume of spirits (30 ml of 40% alc/vol), wine (120 ml of 11% alc/vol) and beer (285 ml of 4.5% alc/vol) equivalent to 10 g of ethanol (a standard drink) were used to prompt reporting of the number of standard drinks usually consumed on each drinking occasion. Reported number of standard drinks were categorized as <2, 2–3, 3.1–6 and >6 standard drinks. Alcohol intake status were categorised as low (< 4 standard drinks for men or <2 standard drinks for women), hazardous (4–6 standard drinks for men or 2–4 standard drinks for women), harmful (consuming at least 6 standard drinks for men or 4 standard drinks for women). The participants were asked about the number of days they usually ate fruit, and the number of days they usually ate vegetables (excluding root plants), in a typical week and how many ‘standard serving’ sizes they usually ate of each on those days. A ‘standard serving’ size of vegetables was defined as a cup of raw vegetables, a half cup of cooked or chopped raw vegetables or half cup of vegetable juice. A ‘standard serving’ size of fruit was defined as a piece of whole of fruit, a half cup of cooked, chopped or processed fruit or half cup of fruit juice and assumed to correspond to 80 gram. Visual aids (show-cards) depicting a ‘standard serving’ size of fruit and vegetables were used to facilitate interviewing. Activity levels were calculated as total time spent on work, transport and leisure time activities of each intensity, weighted by The Global Physical Activity Questionnaire-assigned Metabolic Equivalent Task (MET) energy expenditure ratios per kilogram per hour of 4 for moderate and 8 for vigorous intensity activities. Subjects were categorised as having low (<600MET/week), moderate (>600MET/week), or high (>3000MET/week) activity levels. Measurements of behavioural factors were made and categorised according to recommendations of the WHO [21]

Line 132-133: “The questionnaire was translated into Vietnamese and back-translated to check the accuracy of wording of each item”. The manuscript must be revised to provide a hint at the validity of the so-constructed research tool.

 Our previous publications have focused heavily on the validity of measurements made with the questionnaire. In acknowledgement of this, we have added the following text:

Our previous studies confirmed that the measurements made with the instrument – in aggregate [23], and in respect of tobacco smoking [22], physical activity [24], alcohol intake [25] and fruit and vegetable intake [26] – have validity. 

3. Discussion:

One of the limitations is that the author does not discuss comorbidities related to blood lipid concentrations (hypertension, diabetes, dyslipidemia…).

The comorbidities related to blood lipid concentrations such as high blood pressure and high blood glucose were examined in another of our studies. (Ref: Nga TTT, Blizzard CL, Khue LN, Le Van Ngoc T, Bao TQ, Otahal P, et al. The Interdependence of Blood Pressure and Glucose in Vietnam. High Blood Press Cardiovasc Prev. 2021 Mar;28(2):141-150).

We checked for interdependence of total cholesterol and hypertension or hyperglycaemia, and found no evidence of it. We have added the following text to the Discussion and, at the direction of the reviewer, presented it as a limitation:

This paper does not assess the influence of comorbidities related to blood lipid concentrations, because there was no need to. Elsewhere we have described the inter-dependence of raised blood pressure and elevated blood glucose [39] that should prevent hypertension and hyperglycaemia being modelled in isolation of each other.

Another limitation is related to not including blood lipid-lowering medicine.

To clarify, we have added a sentence to the text of the limitations (line 350). The added text is: 

Moreover, participants were excluded if they reported taking medication to lower TC.

4. Conclusions

You also need to state the implications of the study findings for future research studies, clinical leadership, and policy in the conclusion section

To clarify, we have added the text of the conclusion (line 361-367). The added text is: 

Cholesterol reduction is effective in reducing morbidity and mortality from CVD, and there may be a case for the Government of the Socialist Republic of Vietnam to invest in HRT. Standard clinic guidelines on HRT in Vietnam have not been developed or issued. The gender and age differences in TC levels found in this study suggest that middle-aged Vietnamese women should be the prioritized target for better control of dyslipidaemia and early prevention of cardiovascular disease.

5. References

The references included are relevant for the subject under study, but only show 8/47(17%) references from the last 5 years.

Literature has been revised and updated with the latest publications. The updated references are: 

1. World Health Organization. The global status report on non-communicable diseases. World Health Organization, Geneva; 2010; p.25.

2. Erqou S, Kaptoge S, Perry PL, Di Angelantonio E, Thompson A, White IR, et al. Lipoprotein(a) concentration and the risk of coronary heart disease, stroke, and nonvascular mortality. JAMA. 2009;302(4):412-23.

3. Hajar R. Risk Factors for Coronary Artery Disease: Historical Perspectives. Heart Views. 2017. 18(3): p. 109-114.

4. Virani SS, Alonso A, Aparicio HJ, Benjamin EJ, Bittencourt MS, Callaway CW, et al. Heart Disease and Stroke Statistics-2021 Update: A Report From the American Heart Association. Circulation. 2021. 143(8): p. e254-e743.

5. Carrington MJ and Stewart S. Australia’s cholesterol crossroads: an analysis of 199,331 GP patient records. Baker IDI Heart and Diabetes Institute, Melbourne, Australia; 2011.

6. Woolf K, Reese CE, Mason MP, Beaird LC, Tudor-Locke C, Vaughan LA. Physical activity is associated with risk factors for chronic disease across adult women's life cycle. J Am Diet Assoc, 2008. 108(6): p. 948-59.

7. Balder JW, de Vries JK, Nolte IM, Lansberg, PJ, Kuivenhoven, JA, Kamphuisen, PW. Lipid and lipoprotein reference values from 133,450 Dutch Lifelines participants: Age- and gender-specific baseline lipid values and percentiles. J Clin Lipidol, 2017. 11(4): p. 1055-1064.e6.

8. Ambrož M, de Vries ST, Vart, P, Dullaart, RPF, Roeters van Lennep J, Denig, P, et al. Sex Differences in Lipid Profile across the Life Span in Patients with Type 2 Diabetes: A Primary Care-Based Study. J Clin Med, 2021. 10(8).

9. Gu T, Zhou W, Sun J, Wang J, Zhu D, Bi Y. Gender and age differences in lipid profile among Chinese adults in Nanjing: a retrospective study of over 230,000 individuals from 2009 to 2015. Exp Clin Endocrinol Diabetes. 2018;126(7):429-36.

10. Zhou JL, Lin SQ, Shen Y, Chen Y, Zhang Y, Chen FL. Serum lipid profile changes during the menopausal transition in Chinese women: a community-based cohort study. Menopause. 2010;17(5):997-1003.

11. Gupta R, Sharma M, Goyal NK, Bansal P, Lodha S, Sharma KK. Gender differences in 7 years trends in cholesterol lipoproteins and lipids in India: Insights from a hospital database. Indian J Endocrinol Metab. 2016. 20(2): p. 211-218.

12. Roger VL, Go AS, Lloyd-Jones DM, Adams RJ, Berry JD, Brown TM, et al. Heart disease and stroke statistics-2011 update: a report from the American Heart Association. Circulation. 2011;123(4):e18-e209.

13. Derby CA, Crawford SL, Pasternak RC, Sowers M, Sternfeld B, Matthews KA. Lipid changes during the menopause transition in relation to age and weight: the Study of Women's Health Across the Nation. Am J Epidemiol. 2009;169(11):1352-61.

14. Wang Q, Ferreira DLS, Nelson SM, Sattar N, Ala-Korpela M, Lawlor DA. Lipid changes during the menopause transition in relation to age and weight: the Study of Women's Health Across the Nation. Am J Epidemiol, 2009. 169(11): p. 1352-61.

15. Chae CU, Derby CA. The menopausal transition and cardiovascular risk. Obstet Gynecol Clin North Am. 2011;38(3):477-88.

16. Lee Yh, Lee SG, Lee MH, Kim JH, Lee BW, Kang ES, et al. Serum cholesterol concentration and prevalence, awareness, treatment, and control of high low‐density lipoprotein cholesterol in the Korea National Health and Nutrition Examination Surveys 2008–2010: beyond the tip of the iceberg. J Am Heart Assoc. 2014;3(1):e000650.

17. Kapoor E, Collazo-Clavell ML, Faubion SS. Weight Gain in Women at Midlife: A Concise Review of the Pathophysiology and Strategies for Management. Mayo Clin Proc, 2017. 92(10): p. 1552-1558.

18. Davis SR, Castelo-Branco C, Chedraui P, Lumsden MA, Nappi RE, Shah D, et al. Understanding weight gain at menopause. Climacteric. 2012;15(5):419-29.

19. Ko SH, Kim HS. Menopause-Associated Lipid Metabolic Disorders and Foods Beneficial for Postmenopausal Women. Nutrients, 2020. 12(1).

20. Park JK, Lim YH, Kim KS, Kim SG, Kim JH, Lim HG, et al. Changes in body fat distribution through menopause increase blood pressure independently of total body fat in middle-aged women: the Korean National Health and Nutrition Examination Survey 2007-2010. Hypertens Res, 2013. 36(5): p. 444-9.

21. World Health Organization. WHO STEPS surveillance manual: The WHO STEPwise approach to chronic disease risk factor surveillance. World Health Organization, Geneva 2008 [cited 2018 September 20]. Available from: http://www.who.int/chp/steps/manual/en/.

22. Bui TV, Blizzard L, Luong KN, Truong Nle V, Tran BQ, Ha ST, et al. Declining prevalence of tobacco smoking in Vietnam. Nicotine Tob Res. 2015;17(7):831-8.

23. Bui TV, Blizzard CL, Luong KN, Truong Nle V, Tran BQ, Otahal P, et al. National survey of risk factors for non-communicable disease in Vietnam: prevalence estimates and an assessment of their validity. BMC Public Health. 2016. 16: p. 498.

24. Bui TV, Blizzard CL, Luong KN, Truong Nle V, Tran BQ, Otahal P, et al. Physical activity in Vietnam: estimates and measurement issues. PloS One. 2015;10(10):e0140941

25. Bui TV, Blizzard CL, Luong KN, Van Truong NL, Tran BQ, Otahal P, et al. Alcohol consumption in Vietnam, and the use of 'standard drinks' to measure alcohol intake. Alcohol Alcohol. 2016;51(2):186-95.

26. Bui TV, Blizzard CL, Luong KN, Truong Nle V, Tran BQ, Otahal P, et al. Fruit and vegetable consumption in Vietnam, and the use of a 'standard serving' size to measure intake. Br J Nutr. 2016;116(1):149-57.

27. Choi JS, Song YM, Sung J. Serum total cholesterol and mortality in middle-aged Korean women. Atherosclerosis. 2007;192(2):445-7.

28. Schorr M, Dichtel LE, Gerweck AV, Valera RD, Torriani M, Miller KK, et al. Sex differences in body composition and association with cardiometabolic risk. Biol Sex Differ. 2018. 9(1): p. 28.

29. Schubert CM, Rogers NL, Remsberg KE, Sun SS, Chumlea WC, Demerath EW, et al. Lipids, lipoproteins, lifestyle, adiposity and fat-free mass during middle age: the Fels Longitudinal Study. Int J Obes. 2006;30(2):251-60.

30. Do KA, Green A, Guthrie JR, Dudley EC, Burger HG, Dennerstein L. Longitudinal study of risk factors for coronary heart disease across the menopausal transition. Am J Epidemiol. 2000;151(6):584-93.

31. Cuong DT. Risks and benefits of hormone replacement therapy. Ha Noi: Institute for the Protection of the Mother and Newborn. 2017; [cited 2019 June 3]. Available from: https://www.gfmer.ch/Books/bookmp/143.htm

32. Sohrabji F, Okoreeh A, Panta A. Sex hormones and stroke: beyond estrogens. Horm Behav. 2019;111:87-95.

33. Mauvais-Jarvis F, Clegg DJ, Hevener AL. The role of estrogens in control of energy balance and glucose homeostasis. Endocr Rev. 2013;34(3):309-38.

34. Sai AJ, Gallagher JC, Fang X. Effect of hormone therapy and calcitriol on serum lipid profile in postmenopausal older women: association with estrogen receptor-alpha genotypes. Menopause. 2011;18(10):1101-12.

35. Muga MA, Owili PO, Hsu CY, Chao JC. Association of lifestyle factors with blood lipids and inflammation in adults aged 40 years and above: a population-based cross-sectional study in Taiwan. BMC Public Health. 2019. 19(1): p. 1346.

36. Suliga E, Kozieł D, Cieśla E, Rębak D, Głuszek S. Factors Associated with Adiposity, Lipid Profile Disorders and the Metabolic Syndrome Occurrence in Premenopausal and Postmenopausal Women. PLoS One. 2016. 11(4): p. e0154511.

37. Kelley GA, Kelley KS, Tran ZV. Exercise, lipids, and lipoproteins in older adults: a meta-analysis. Prev Cardiol. 2005;8(4):206-14.

38. Aadahl M, von Huth Smith L, Pisinger C, Toft UN, Glumer C, Borch-Johnsen K, et al. Five-year change in physical activity is associated with changes in cardiovascular disease risk factors: the Inter99 study. Prev Med. 2009;48(4):326-31.

39. Nga TTT, Blizzard CL, Khue LN, Le Van Ngoc T, Bao TQ, Otahal P, et al. The Interdependence of Blood Pressure and Glucose in Vietnam. High Blood Press Cardiovasc Prev. 2021 Mar;28(2):141-150.

Attachment Submitted filename: Response to Reviewers.docx Click here for additional data file.

# Peer review: Decision Letter 1

**DOI:** 10.1371/journal.pone.0256589.r003

**Authors:** Lee-Ling Lim

11 Aug 2021

Sex differences in total cholesterol of Vietnamese adults

PONE-D-20-37824R1

Dear Dr. Blizzard,

We’re pleased to inform you that your manuscript has been judged scientifically suitable for publication and will be formally accepted for publication once it meets all outstanding technical requirements.

Within one week, you’ll receive an e-mail detailing the required amendments. When these have been addressed, you’ll receive a formal acceptance letter and your manuscript will be scheduled for publication.

An invoice for payment will follow shortly after the formal acceptance. To ensure an efficient process, please log into Editorial Manager at http://www.editorialmanager.com/pone/, click the 'Update My Information' link at the top of the page, and double check that your user information is up-to-date. If you have any billing related questions, please contact our Author Billing department directly at authorbilling@plos.org.

If your institution or institutions have a press office, please notify them about your upcoming paper to help maximize its impact. If they’ll be preparing press materials, please inform our press team as soon as possible -- no later than 48 hours after receiving the formal acceptance. Your manuscript will remain under strict press embargo until 2 pm Eastern Time on the date of publication. For more information, please contact onepress@plos.org.

Kind regards,

Lee-Ling Lim

Academic Editor

PLOS ONE

Additional Editor Comments (optional):

Reviewers' comments:

Reviewer's Responses to Questions

**Comments to the Author**

1. If the authors have adequately addressed your comments raised in a previous round of review and you feel that this manuscript is now acceptable for publication, you may indicate that here to bypass the “Comments to the Author” section, enter your conflict of interest statement in the “Confidential to Editor” section, and submit your "Accept" recommendation.

Reviewer #1: All comments have been addressed

Reviewer #2: (No Response)

**********

2. Is the manuscript technically sound, and do the data support the conclusions?

The manuscript must describe a technically sound piece of scientific research with data that supports the conclusions. Experiments must have been conducted rigorously, with appropriate controls, replication, and sample sizes. The conclusions must be drawn appropriately based on the data presented. 

Reviewer #1: Yes

Reviewer #2: Yes

**********

3. Has the statistical analysis been performed appropriately and rigorously? 

Reviewer #1: Yes

Reviewer #2: Yes

**********

4. Have the authors made all data underlying the findings in their manuscript fully available?

The PLOS Data policy requires authors to make all data underlying the findings described in their manuscript fully available without restriction, with rare exception (please refer to the Data Availability Statement in the manuscript PDF file). The data should be provided as part of the manuscript or its supporting information, or deposited to a public repository. For example, in addition to summary statistics, the data points behind means, medians and variance measures should be available. If there are restrictions on publicly sharing data—e.g. participant privacy or use of data from a third party—those must be specified.

Reviewer #1: Yes

Reviewer #2: (No Response)

**********

5. Is the manuscript presented in an intelligible fashion and written in standard English?

PLOS ONE does not copyedit accepted manuscripts, so the language in submitted articles must be clear, correct, and unambiguous. Any typographical or grammatical errors should be corrected at revision, so please note any specific errors here.

Reviewer #1: Yes

Reviewer #2: Yes

**********

6. Review Comments to the Author

Please use the space provided to explain your answers to the questions above. You may also include additional comments for the author, including concerns about dual publication, research ethics, or publication ethics. (Please upload your review as an attachment if it exceeds 20,000 characters)

Reviewer #1: (No Response)

Reviewer #2: (No Response)

**********

7. PLOS authors have the option to publish the peer review history of their article (what does this mean?). If published, this will include your full peer review and any attached files.

If you choose “no”, your identity will remain anonymous but your review may still be made public.

**Do you want your identity to be public for this peer review?** For information about this choice, including consent withdrawal, please see our Privacy Policy.

Reviewer #1: No

Reviewer #2: No

# Editor report: Acceptance letter

**DOI:** 10.1371/journal.pone.0256589.r004

**Authors:** Lee-Ling Lim

13 Aug 2021

PONE-D-20-37824R1 

Sex differences in total cholesterol of Vietnamese adults 

Dear Dr. Blizzard:

I'm pleased to inform you that your manuscript has been deemed suitable for publication in PLOS ONE. Congratulations! Your manuscript is now with our production department. 

If your institution or institutions have a press office, please let them know about your upcoming paper now to help maximize its impact. If they'll be preparing press materials, please inform our press team within the next 48 hours. Your manuscript will remain under strict press embargo until 2 pm Eastern Time on the date of publication. For more information please contact onepress@plos.org.

If we can help with anything else, please email us at plosone@plos.org. 

Thank you for submitting your work to PLOS ONE and supporting open access. 

Kind regards, 

PLOS ONE Editorial Office Staff

on behalf of

Dr. Lee-Ling Lim 

Academic Editor

PLOS ONE
